# Anti-malarial drugs: how effective are they against *Plasmodium falciparum *gametocytes?

**DOI:** 10.1186/1475-2875-11-34

**Published:** 2012-02-06

**Authors:** Christopher L Peatey, Didier Leroy, Donald L Gardiner, Katharine R Trenholme

**Affiliations:** 1 Malaria Biology Laboratory, Queensland Institute of Medical Research, 300 Herston Rd, Herston 4006, Queensland, Australia; 2 School of Molecular and Microbial Sciences University of Queensland, Herston, Queensland, Australia; 3 Medicines for Malaria Venture, Route de Pré-Bois 20, PO Box 1826, 1215 Geneva 15, Switzerland; 4 School of Biomolecular and Physical Sciences, Griffith University, Herston, Queensland, Australia; 5 School of Medicine, University of Queensland, Herston, Queensland, Australia

**Keywords:** *Plasmodium falciparum*, Gametocyte, Anti-malarial drugs

## Abstract

**Background:**

Recent renewed emphasis on the eradication of malaria has highlighted the need for more tools with which to achieve this ambitious goal. One high priority area is the need to determine the gametocytocidal activity of both currently used anti-malarial drugs and those in the development pipeline. However, testing the activity of compounds against *Plasmodium falciparum *gametocytes is technically challenging both *in vivo *and *in vitro*.

**Methods:**

Here the use of a simple robust assay to screen a panel of currently used and experimental anti-malarial drugs against mature *P. falciparum *gametocytes is described.

**Results:**

Eight of 44 compounds tested reduced gametocyte viability by at least 50% and three showed IC_50 _values in nM range.

**Conclusions:**

There is a need to identify new compounds with activity against late stage gametocytes and the information provided by this *in vitro *assay is a valuable first step, which can guide future clinical studies.

## Background

Gametocytes are the sexual stage of the malaria parasite, which develop in red blood cells and are essential for transmission to the mosquito vector. It has long been recognized that patients treated for malaria should be cleared of gametocytes in order to prevent them transmitting the infection to others [1]. This is particularly challenging in the case of *Plasmodium falciparum *infections as gametocytes of this species have a much longer lifespan than asexual stages. Late-stage gametocytes (stages IV-V) are more resistant to anti-malarial drugs and metabolic inhibitors [2] than early-stage gametocytes or asexual stage parasites. Primaquine is currently the only licensed anti-malarial drug that is effective against late stage *P. falciparum *gametocytes but has a number of drawbacks including its propensity to cause acute haemolysis in individuals with glucose-6-phosphate dehydrogenase (G6PD) deficiency.

With malaria eradication back on the global health agenda there is renewed emphasis on the identification of new and novel agents that can eliminate late-stage gametocytes in the patient's circulation and therefore block transmission of the parasite from its human host to the mosquito vector. However, evaluating the activity of promising anti-malarial drugs against *Plasmodium *gametocytes is difficult even *in vitro*. To this end a simple medium-high throughput assay suitable for assessing the potential of new and novel anti-gametocyte drugs has recently been described [3].

Here a modification of this innovative assay system is used to evaluate the activity of a panel of 44 compounds (comprising currently used and experimental anti-malarial drugs) against mature *P. falciparum *gametocytes.

## Methods

Dixon *et al. *have previously reported the development of an assay utilizing a green fluorescent protein chimera of the early sexual blood stage protein Pfs16 as a marker for commitment to gametocytogenesis [4]. This reporter system allows accurate identification of gametocytes well before they are morphologically distinguishable from asexual parasites and allows for the isolation of large numbers of pure gametocytes.

### Production of late stage gametocytes

Mature stage V gametocytes were obtained as described previously [3], briefly, 30 mL of synchronous ring stage cultures of the transgenic parasite line 3D7GFP16B were established at 1-2% parasitaemia. The parasites were grown under standard conditions [5] through one complete invasion cycle and gametocyte production induced by the addition of conditioned media to trophozoite stage parasites [6]. The parasites were grown through one further invasion cycle and gametocytes were separated from asexual parasites and uninfected red blood cells using a modified Percoll step gradient [7]. These gametocyte-enriched cultures were sorted and collected using a BD FACSAria II cell sorter (BD Biosciences). The gametocytes were sorted based on expression of the GFP reporter gene to give only stage I/II gametocytes which were matured to stage V *in vitro *under standard culture conditions. This maturation takes between 5 and 7 days and was monitored daily by examination of Giemsa stained thin smears.

### Drug assay

Approximately 5,000 gametocytes were dispensed into each well of a 96-well plate. The test compounds were added to give a final volume of 100 μl and the plate incubated at 37°C for 24 hours under standard culture conditions. BacTiter-Glo reagents (Promega G8231) were added to a final volume of 200 μl and the assay read using a GloMax^® ^96 Microplate Luminometer [Cat.# E6501] with an integration constant of 0.5 seconds. BacTitre-Glo is a homogenous ATP-based bioluminescent assay for detecting and quantifying viable microbial cell numbers. The assay uses a single reagent to release and measure the ATP contained in the cells. The presence of ATP indicates live cells and when compared to a known standard can provide quantitative data on the gametocytocidal effect of compounds.

All compounds were dissolved in DMSO and tested in triplicate on three separate occasions at a final concentration of 10 μM (1% DMSO). The assay included a positive control for gametocytocidal effect that consisted of gametocytes lysed with H_2_O_2 _and a negative control made of standard culture media with 1% DMSO (final concentration of solvent in all samples tested).

### Data analysis

Read-outs from drug-treated wells were compared to those of control wells and percentage inhibition calculated. IC_50 _values were determined using Graphpad Prism.

## Results

**Figure 1:**
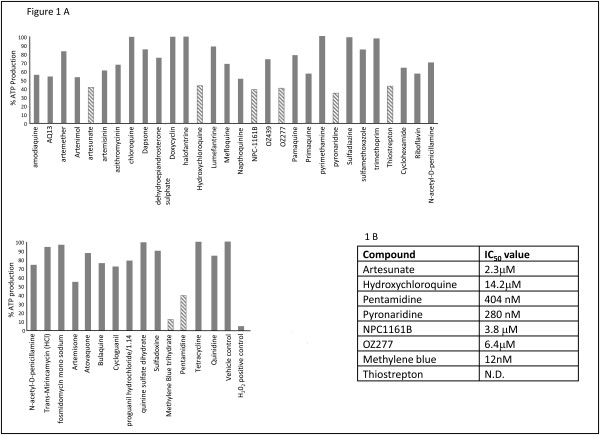
**A: The activity of a panel of currently used and experimental anti-malarial drugs against late stage *Plasmodium falciparum *gametocytes**. Bars with diagonal lines indicate compounds which resulted in a greater than 50% reduction in ATP production as compared to the control. All drugs were tested at a concentration of 10 μM. **B: **IC_50 _values were determined for compounds which demonstrated a greater than 50% reduction in ATP production.

A panel of 44 compounds, provided by the Medicines for Malaria Venture, was made up of both currently used and experimental anti-malarial drugs. All compounds were tested at a single concentration of 10 μM in a blinded study for activity against late stage *P. falciparum *gametocytes. Compounds were unblinded following data analysis (Figure 1A).

Of the 44 compounds tested eight demonstrated a reduction in ATP production greater than 50%, indicating that these compounds reduced gametocyte viability by at least 50%. Methylene blue was the most active compound, giving an 88% reduction in ATP production compared to controls; a further seven compounds showed inhibition of ATP production ranging from 57% (hydroxychloroquine) to 66% (pyronaridine). Of the remaining 39 compounds, several showed no inhibition of ATP production, indicating they have no activity against mature gametocytes; these include the currently used anti-malarial drugs chloroquine, doxycyclin and quinine.

Compounds which demonstrated a greater than 50% reduction in ATP production also underwent dose response testing in an 11-point dilution curve at concentrations ranging from 5 μM to 0.5 nM. IC_50 _values were determined using Graphpad Prism and are shown in Figure 1B. Thiostrepton was excluded from this analysis due to insufficient compound for testing at a range of concentrations.

Three compounds had IC_50 _values in the nM range; methylene blue had the lowest at IC_50 _value at 12 nM, and was followed by pyronaridine (280 nM) and pentamidine (404 nM).

## Discussion

Ideal drugs and combination therapies for malaria eradication campaigns should clear asexual parasitaemia, reducing clinical symptoms as quickly as possible and prevent transmission of the sexual stages. However, gametocytes are refractory to most currently used anti-malarials and this lack of drugs with activity against late-stage *P. falciparum *gametocytes makes identification of new compounds with such activity a high priority. Indeed, the malERA Consultative Group on Drugs [8] recently identified the need to determine the gametocytocidal activity of both currently used anti-malarial drugs and those in the development pipeline as a high priority, alongside the need to develop new drugs with the ability to kill or prevent development of gametocytes.

Here a simple, rapid *in vitro *assay is used to undertake a comprehensive study into the effect of currently used and experimental anti-malarial drugs on late stage gametocytes. Eight out of 44 compounds tested reduced gametocyte viability by at least 50%. Three of these were currently used anti-malarial drugs, hydroxychloroquine, artesunate, pyronaridine and three were anti-malarial drugs currently in the development pipeline: NPC1161B, OZ277 and methylene blue. Two additional compounds, pentamidine: an anti-microbial effective in treatment of leishmaniasis and *Trypanosoma brucei*, and thiostrepton, a complex natural product derived from Streptomyces and mainly used in veterinary medicine, are unlikely to be suitable for mass use as anti-gametocyte agents. The finding, that artesunate has demonstrated activity against mature stage gametocytes, confirms the findings of other groups but this does not appear to translate into a significant clinical effect against mature stage gametocytes in patients [9]. The most likely explanation is that *in vitro *testing cannot take into account *in vivo *pharmacokinetics, and artesunate has a relatively short half-life *in vivo *[10]. However, the *in vitro *activity of methylene blue has been shown to translate into strong gametocytocidal activity against *P. falciparum in vivo *[11]. Primaquine has been reported to kill mature-stage gametocytes, however, in this study primaquine showed a 43% inhibition in ATP production but the *in vivo *effect is likely to be much greater as it is thought that one or more metabolites of primaquine are responsible for its observed gametocytocidal activity *in vivo *[12]

Testing the activity of compounds against *P. falciparum *gametocytes is difficult both *in vivo *and *in vitro*. An advantage of the *in vitro *testing described here is that it allows compounds to be assessed in the absence of confounding host factors but has the disadvantage that it is not suitable for testing anti-folate drugs such as sulphadoxine and proguanil, because the transgenic parasite clone used in the study has altered sensitivity to folate antagonists [4]. Pyrimethamine was tested as part of this blinded study and as expected no reduction in ATP production was seen. However, the creation of transgenic parasite lines with alternative drug selection cassettes is possible and would overcome this limitation. It is also feasible that some compounds may inhibit the detection of ATP, resulting in false positives, so as with all such assays additional orthogonal screening is still recommended.

The relatively small number of compounds shown to have significant anti-gametocyte activity in this study highlights the lack of compounds with activity against this important parasite life cycle stage and emphasizes the need for continued research in this area.

## Conclusion

This study reports the use of a novel *in vitro *assay to test the activity of a panel of currently used and experimental anti-malarial drugs against late stage *P. falciparum *gametocytes. Only eight out of 44 compounds tested reduced gametocyte viability by at least 50%, highlighting the need to develop novel compounds with activity against this key life cycle stage. When used as part of a screening programme the information provided by this assay is a valuable first step, which can guide future clinical studies.

## Competing interests

The authors declare that they have no competing interests.

## Authors' contributions

DLG, KRT and DL conceived the study, DLG, KRT and CLP designed the study, undertook the experimental procedures and data analysis. All authors participated in interpretation of the data. KRT drafted the final manuscript, which was read and approved by all authors.
